# miRNAs in the vitreous humor of patients affected by idiopathic epiretinal membrane and macular hole

**DOI:** 10.1371/journal.pone.0174297

**Published:** 2017-03-22

**Authors:** Andrea Russo, Marco Ragusa, Cristina Barbagallo, Antonio Longo, Teresio Avitabile, Maurizio G. Uva, Vincenza Bonfiglio, Mario D. Toro, Rosario Caltabiano, Cesare Mariotti, Francesco Boscia, Mario Romano, Cinzia Di Pietro, Davide Barbagallo, Michele Purrello, Michele Reibaldi

**Affiliations:** 1 Department of Ophthalmology, University of Catania, Catania, Italy; 2 Molecular, Genome and Complex Systems BioMedicine Unit, Department of Biomedical Sciences and Biotechnology, University of Catania, Catania, Italy; 3 Department Gian Filippo Ingrassia, Unità di Anatomia Patologica, University of Catania, Catania, Italy; 4 Department of Ophthalmology, University of Ancona, Ancona, Italy; 5 Department of Ophthalmology, University of Sassari, Sassari, Italy; 6 Department of Ophthalmology, Second University of Napoli, Napoli, Italy; University College London, UNITED KINGDOM

## Abstract

**Purpose:**

The aim of the present study was to assess the expression of miRNAs in the Vitreous Humor (VH) of patients with Macular Hole (MH) and Epiretinal Membrane (ERM) compared to a control group.

**Methods:**

In this prospective, comparative study, 2-ml of VH was extracted from the core of the vitreous chamber in consecutive patients who underwent standard vitrectomy for ERM and MH. RNA was extracted and TaqMan^®^ Low Density Arrays (TLDAs) were used to profile the transcriptome of 754 miRNAs. Results were validated by single TaqMan^®^ assays. Finally, we created a biological network of differentially expressed miRNA targets and their nearest neighbors.

**Results:**

Overall 10 eyes with MH, 16 eyes with idiopathic ERM and 6 controls were enrolled in the study. Profiling data identified 5 miRNAs differentially expressed in patients affected by MH and ERM with respect to controls. Four were downregulated (miR-19b, miR-24, miR-155, miR-451) and 1 was downregulated (miR-29a); TaqMan^®^ assays of the VH of patients affected by MH and ERM, with respect to controls, showed that the most differentially expressed were miR-19b (FC -9.13, p:<0.00004), mir-24 (FC -7.52, p:<0.004) and miR-142-3p (FC -5.32, p:<0.011). Our network data showed that deregulation of differentially expressed miRNAs induces an alteration of several pathways associated with genes involved in both MH and ERM.

**Conclusion:**

The present study suggests that disregulation of miR-19b, miR-24 and miR-142-3p, might be related to the alterations that characterize patients affected by MH and ERM.

## Introduction

Vitreo maculopathies are characterized by traction exerted on the macula generated by the vitreous and the inner limiting membrane of the retina. Traction arising from vitreomacular adhesions can be tangential or perpendicular to the retinal surface. Both conditions might determine features of clinical pathologies including epiretinal membrane (ERM) and macular hole (MH); in these diseases epiretinal cell proliferations and fibrosis are essential parts of the pathogenesis [1–2].

Most MHs are idiopathic, however, they can also be found in highly myopic eyes, or after a ocular trauma [3]. Pseudoholes, secondary to ERMs, should be differentiated from full thickness MHs [4]. ERMs are detected in about two-thirds of eyes affected by MHs [5–6].

ERM can be idiopathic or secondary to several vitreoretinal diseases and is characterized by cellular contraction after fibrocellular proliferation on the inner limiting membrane. Posterior vitreous detachment (PVD) can injure the internal limiting membrane, allowing movement of glial cells to the retinal surface. Furthermore, an incomplete PVD might provide appropriate conditions for fibrocellular proliferation in the area between the vitreous and the retina [7]. In the process of ERM formation, extracellular matrix, cytokines and growth factors are involved in cellular signal transmission and in tissue changes [8].

Some studies have shown that a number of regulatory factors have also significant effects on fibrosis and may be related to its inter-organ variability [9].

miRNAs are small, non-coding RNAs with a strictly regulated biogenesis. This is combined with an extremely flexible and sophisticated regulatory function, allowing simultaneous targeting of multiple mRNAs coding for proteins involved in different, crucial biological pathways of specific cell types and tissues [10].

miRNAs exert control over cellular processes such as differentiation and proliferation acting on various targets [11], and may play the role of conductors in the pathogenesis of fibrosis [12].

miRNA alterations are common in different fibrotic disorders such as systemic sclerosis [13], liver cirrhosis [14], cardiac fibrosis [15–16], chronic kidney disease [17], and idiopathic pulmonary fibrosis [18].

The discovery, in 2008, of miRNAs circulating in human blood opened new intriguing perspectives in molecular diagnosis [19,20]. Circulating miRNAs have been shown to be present in several biological fluids (e.g., serum, plasma, cerebrospinal fluid, vitreous humor) in a stable form that prevents their digestion by RNases. However, little is known about the origin and function of circulating miRNAs. One of the most fascinating hypotheses is that extracellular miRNAs may work as mediators of cell-cell communication: specific miRNAs are selectively secreted by donor cells to be functionally transferred to recipient cells [21,22]. Currently, two major release mechanisms of circulating miRNAs have been proposed: (i) secretion of miRNAs stored inside microvesicles or exosomes [23]; (ii) secretion of miRNAs complexed to ribonucleoproteins [24]. Since concentration of circulating miRNAs is related to the physiological and pathological condition of patients, it is not surprising that they have already been exploited as molecular biomarkers for neoplastic and degenerative diseases.

Our group previously showed the presence of miRNAs in the Vitreous Humor (VH) and that the expression of circulating miRNAs in VH is altered in different eye diseases [25,26]. Recent reviews have addressed the role of miRNAs in fibrosis with a focus on organ-specific miRNA alteration [27–30] and both pathologies are caused by mechanisms related to fibrosis.

The aim of the present study was to assess the expression of miRNAs in the VH of patients with MH and ERM compared to a control group.

## Methods

This prospective, comparative study included all consecutive eyes of patients who underwent vitrectomy at the Ophthalmic Clinic of the University of Catania, for MH and ERM, between September 2015 and April 2016. Controls were all consecutive eyes of patients, matched by age and sex, who underwent vitrectomy for primary symptomatic idiopathic floaters in the same period.

Floaters represent the least compromised condition for eyes to undergo vitreous surgery, since it is not possible to remove the vitreous from living healthy subjects.

The study adhered to the tenets of the Declaration of Helsinki and was approved by the Local Ethics Research Committee (“Comitato Etico Catania1”). Before the procedures, written informed consent was obtained from all participants in the study.

All eyes had idiopathic MHs with a minimum size > 250 μm [31] and idiopathic fovea-involving ERM, with prominent thickening of the inner retinal layer [32]. Both MHs and ERM were diagnosed ophthalmoscopically and with a Spectralis Optical Coherence Tomography (OCT) examination (Heidelberg Engineering, Heidelberg, Germany).

We excluded from our study patients with diabetes, cardiovascular failure, autoimmune diseases, renal or hepatic failure, Alzheimer’s, and Parkinson’s disease. We also excluded patients who had undergone previous ocular surgical procedures, affected by glaucoma, uveitis, diabetic retinopathy and other retinopathies, ocular trauma, and any ocular tumor, as the amount of vitreal miRNAs could be modified, depending on the diseases of the eye [25].

All patients underwent a 3-port 25-gauge vitrectomy performed by the same surgeon (M.R.) under local anesthesia. The Resight 700 (Carl Zeiss Meditec AG, Jena, Germany) wide-angle viewing system or the Binocular Indirect Ophthalmol Microscope wide-angle viewing system (BIOM; Oculus Inc, Wetzlar, Germany) were used. Sclerotomies were placed at 3.5 mm to the limbus and performed in a 30° fashion, parallel to the limbus [33]. With closed infusion, a 2-ml vitreous specimen was extracted from the core of the vitreous cavity into a syringe using a three-way tap. The extracted vitreous was then placed in a sterile container, and the vitrectomy continued as normal. Samples were centrifuged at 700 ×g for 10’ to pellet and eliminate any circulating cells and finally stored at -80°C until analysis.

### RNA extraction

Using a Qiagen miRNeasy Mini Kit (Qiagen, GmbH, Hilden, Germany), RNA was extracted from 500-μl vitreous samples. RNA was eluted in a 30 μl volume of elution buffer with two repeated steps in the same collection tube. RNAs were quantified by fluorometery (Qubit, Invitrogen) and spectrophotometery (GeneQuant Pro, BioChrom Ltd, Cambridge, UK).

### miRNA expression analysis

According to the manufacturer’s instructions, 4.5 μl of vitreal RNAs were retrotranscribed and preamplified to profile the transcriptome of 754 miRNAs, and then loaded on TaqMan^®^ Low Density Arrays (TLDAs) TaqMan^®^ Human MicroRNA Array v3.0 A and B (Applied Biosystems, Foster City, CA, USA). PCRs on TLDAs were conducted on a 7900HT Fast Real Time PCR System (Applied Biosystems). Results were validated by single TaqMan^®^ assays and TaqMan^®^ Universal Master mix II (Life Technologies, Italy) using 20 ng of vitreal RNA, according to the manufacturer’s instructions.

### Statistical analysis

To obtain an accurate miRNA profiling, we used the global median normalization method, as previously reported for the same kind of analysis [26]. By this approach, we identified small RNAs that presented an expression profile near to the median of TLDAs, i.e. snRNA U6 and miR-223. Accordingly, they were then used as reference genes for analysis of TLDAs. By Significance Analysis of Microarrays (SAM), differentially expressed miRNAs were identified, computed by Multi experiment viewer v4.8.1, by applying a two-class unpaired test among ΔCts and using a p-value based on 100 permutations; imputation engine: K-nearest neighbors (10 neighbors); false discovery rate < 0.05 was used as correction for multiple comparisons. The 2−ΔΔCT method was used to calculate the Expression fold changes (FC). SnRNA U6 was used as reference gene for single TaqMan^®^ validation assays. The unpaired t-test (p < 0.05) was applied to statistically evaluate the expression differences between patients and healthy controls by single TaqMan^®^ validation assays. Statistical significance was established at a p-value > 0.05. ΔCts for differentially expressed miRNAs with respect to endogenous control snRNA U6 were used to generate a receiver operating characteristic (ROC) curve (MedCalc 15.11.4). The area under the curve (AUC) and 95% confidence intervals were calculated to assess the accuracy of each parameter (sensitivity and specificity) and to find an appropriate cut-off point. Statistical significance of ROC curves was established at a p-value > 0.05.

### Network construction and analysis

To evaluate the biological meaning of differentially expressed miRNAs, we retrieved their experimentally validated targets from miRTarBase (http://mirtarbase.mbc.nctu.edu.tw/). To statistically enrich the gene signaling regulated by differentially expressed miRNAs, we built a network based on interactions between differentially expressed miRNA targets and their nearest neighbors. This network was generated using Cytoscape v2.8.3 (www.cytoscape.org/) and MiMI plugin (http://mimiplugin.ncibi.org/). We determined statistical over-representation of pathways by using the FatiGO tool (http://babelomics3.bioinfo.cipf.es) on the genes from the previously generated network that screened Gene Ontology (GO), KEGG and Reactome databases. Statistical over-representation was calculated by using Fisher's exact test; Benjamini & Hochberg FDR Correction; p ≤ 0.005. The over-represented pathways in this analysis were associated with dysregulated genes involved in ERM and MHs, as reported in the literature (https://www.ncbi.nlm.nih.gov/pubmed/).

## Results

Comparison of vitreal miRNA profiles from patients affected by MHs and ERM with those of controls was performed.

VH samples were extracted from 32 eyes after surgery: 10 eyes with MHs) (mean age 60±6), 16 eyes affected by ERM (mean age 59±5) and 6 controls (mean age 60±7). Eighteen (56%) were male and 14 (44%) female.

Using TLDA technology, we determined the profiles of 754 miRNAs in the VH, from 4 MHs, 4 ERMs and 4 controls (Ct data are reported in supplementary material 1). The comparison of miRNA profiles in the VH of different patient classes by SAM statistical method showed 9 differentially expressed miRNAs (Table 1).

**Table 1:** Nine differentially expressed miRNAs.

miRNAs	FC ERM + MH vs Cs	FC ERM vs MH
miR-19b	-5	NDE
miR-24	-3	NDE
miR-29a	3	-5.16
miR-30a-3p	NDE	2.42
miR-142-3p	NDE	-4.2
miR-155	-4.2	NDE
miR-451	-5	NDE
miR-574-3p	NDE	4.13
miR-1290	NDE	3.3

Differentially Expressed vitreal miRNAs by TLDAs (TaqMan Low Density Arrays) in the vitreous humor of patients affected by macular hole and epiretinal membrane with respect to controls and comparison between pathological classes.

All Differentially Expressed miRNAs showed a false discovery rate < 0.05 based on Significance Analysis of Microarray test

FC, Fold Change; Cs, Controls; NDE, Not Differentially Expressed.

More specifically, we found 4 downregulated miRNAs (miR-19b, miR-24, miR-155, miR-451) and 1 upregulated miRNA (miR-29a) in patients affected by MH and ERM with respect to controls; while, 2 downregulated miRNA (miR-29a, miR-142-3p) and 3 upregulated miRNAs (miR-30a-3p, miR-574-3p, miR-1290) were found by comparing ERMs to MHs (Table 1). Profiling data showed that 4/5 of differentially expressed miRNAs had a negative FC, suggesting a general trend of downregulation of circulating miRNAs in the VH of eyes with MH and ERM with respect to controls. miR-30a-3p, miR-574-3p, miR-1290 were statistically more abundant in the VH of ERM patients than MH patients.

### Validation by single TaqMan^®^ assays

**Fig 1:**
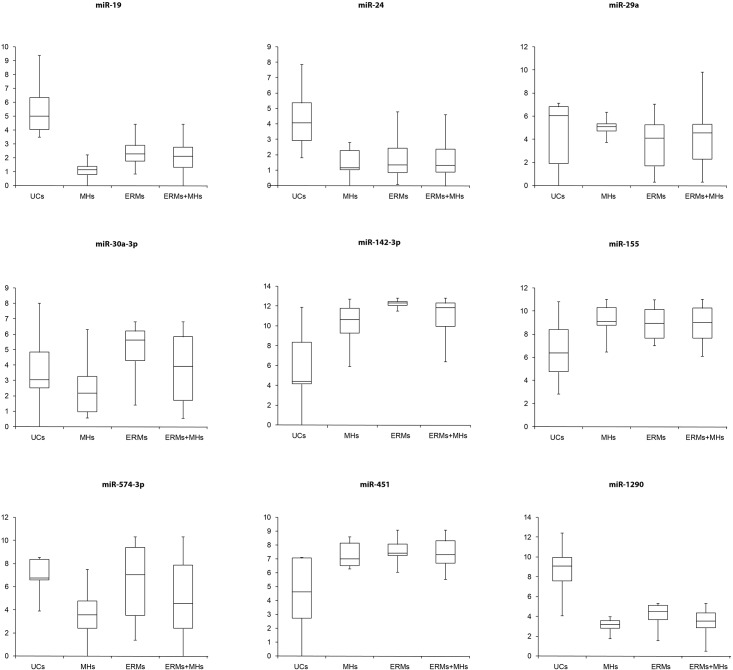
Box Plots of differentially expressed miRNAs. Box Plots from Single TaqMan^®^ Assays on TaqMan^®^ Low Density Arrays (TLDA) of differentially expressed miRNAs. Validation by single TaqMan^®^ assays of differentially expressed miRNAs identified by TLDAs in the vitreous humor of all the patients and controls. Values on the y-axis are reported as ΔCt × (−1).

Expression of differentially expressed miRNAs identified by TLDAs was confirmed by single TaqMan^®^ assays in the VH of all the patients and controls (Table 2) (Fig 1) (S1 File).

**Table 2:** Differentially expressed vitreal miRNAs.

DE miRNAs	ERM + MH vs Cs		MH vs Cs		ERM vs Cs		ERM vs MH	
	*FC*	t-test	*FC*	t-test	*FC*	t-test	*FC*	t-test
miR-19b	-9.13	0.00004	-14.1	0.002	-6.29	0.0004	2.24	0.046
miR-24	-7.52	0.004	-11.58	0.012	-6.38	0.016	-	NS
miR-29a	-	NS	-	NS	-	NS	-1.94	0.031
miR-30a-3p	-	NS	-	NS	-	NS	11.08	0.026
miR-142-3p	5.32	0.011	5.81	0.048	4.83	0.021	3.31	0.013
miR-155	6.88	0.018	7.21	0.015	6.45	0.019	-	NS
miR-451	6.52	0.041	5.35	0.045	6.96	0.038	-	NS
miR-574-3p	-	NS	-7.55	0.018	-	NS	7.48	0.047
miR-1290	-6.29	0.036	-8.14	0.031	-5.74	0.042	-	NS

Differentially Expressed vitreal miRNAs by single TaqMan^®^ assays in the vitreous humor of patients affected by macular hole and epiretinal membrane with respect to controls and comparison between pathological classes.

t test: significant p-value < 0.05.

FC, Fold Change; Cs, Controls; NS, Not Significant.

The downregulation of miR-19b in the VH of pathological patients with respect to controls was statistically confirmed by applying the t-test, but we also detected its upregulation in ERMs with respect to MHs (Table 2) (Fig 1). The downregulation of miR-24 and miR-1290 and the upregulation of miR-142-3p, miR-155 and miR-451 in ERMs and MHs compared to controls was also validated (Table 2) (Fig 1). Moreover, miR-142-3p, miR-30a-3p, miR-574-3p were statistically more abundant in ERMs with respect to MHs; while miR-29a was downregulated in the same comparison (Table 2) (Fig 1). We obtained no statistical validation on the upregulation of miR-29a in patients affected by MHs and ERMs with respect to controls.

### Network and pathway enrichment analysis

**Fig 2:**
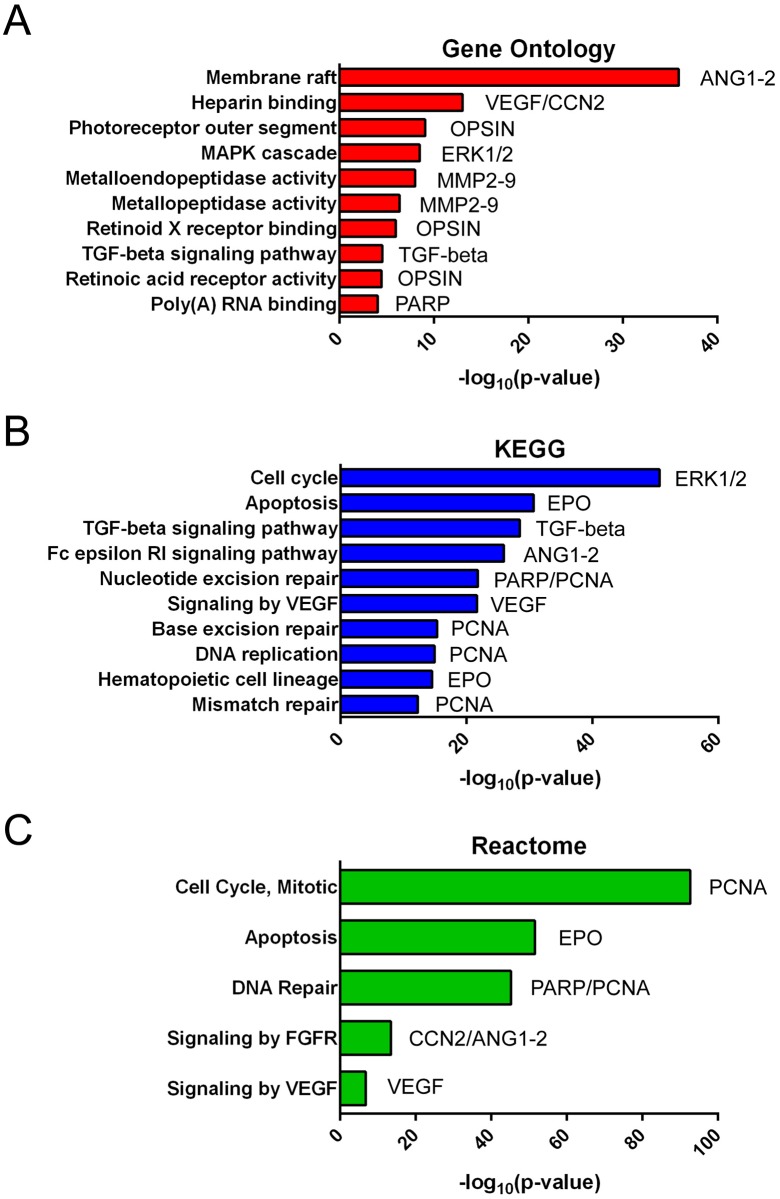
Biological processes controlled by miR-19b, miR-24 and miR-142-3p network. Overrepresented biological functions from a molecular network built on validated targets of differentially expressed miRNAs, retrieved from different annotation databases (GO, KEGG, Reactome). On the left of each histogram the overrepresented pathways are reported, while on the right the corresponding associated genes to MHs and ERMs based on literature data are shown. Data are plotted as—log10 of p-values for each biological process.

To understand the potential functional effect of deregulation of the 9 differentially expressed miRNAs we created a biological network based on differentially expressed miRNA targets and their nearest neighbors. Considering all network nodes, we analyzed the statistical over-representation of biological pathways from various databases (i.e. Reactome, KEGG, and GO) against the whole genome (Fig 2).

Our data showed that observed miRNA deregulation could induce an alteration of several pathways recently associated with genes involved in vitreoretinal diseases, such as MHs, and ERMs.

### ROC curves

**Fig 3:**
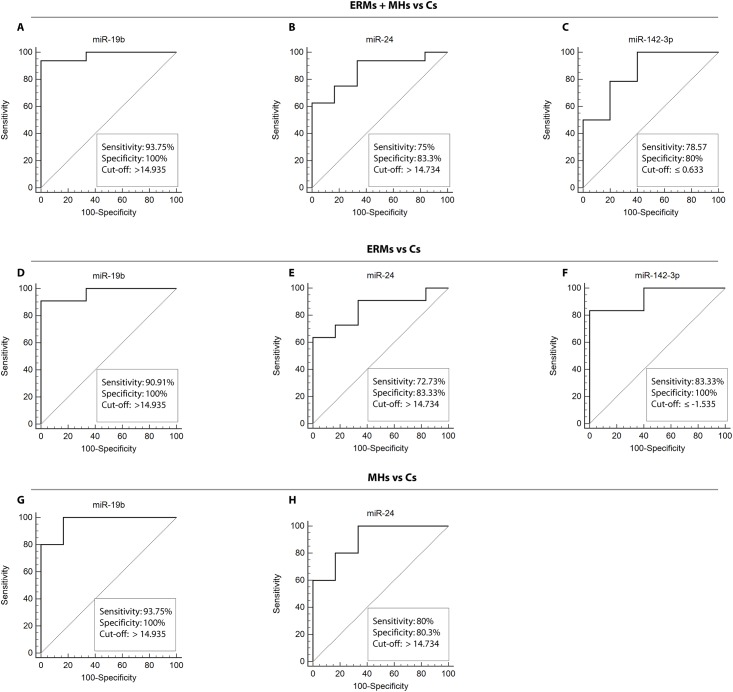
Receiver Operator Characteristic (ROC) curves for vitreal miR-19b, miR-24 and miR-142-3p in patients affected by MHs and ERMs. ROC curves of miR-19b (A), miR-24 (B), miR-142-3p (C) DCts in comparison with ERMs + MHs vs controls; miR-19b (D), miR-24 (E), miR-142-3p (F) DCts in comparison with ERMs vs controls; miR-19b (D), miR-24 (E) in comparison with MHs vs controls. Curves represent DCts calculated by using U6 as endogenous control.

To evaluate the discriminating power of the differentially expressed vitreal miRNAs as potential markers of ERMs and MHs, we computed the ROC curves for each type of comparison: ERMs + MHs vs controls, ERMs vs controls, MHs vs controls, ERMs vs MHs. Our analysis showed significant results for just three of the 9 differentially expressed miRNAs: miR-19b, miR-24 and miR-142-3p. More specifically, we found for ERMs + MHs vs controls that miR-19b had an AUC of 0.979 (95% CI, 0.810–1; p< 0.0001) with 93.75% sensitivity and 100% specificity (DCt cut-off value: >14.935); miR-24 showed an AUC of 0.865 (95% CI, 0.652–0.971; p< 0.0001) with 75% sensitivity and 83.33% specificity (DCt cut-off value: > 14.734); miR-142-3p had an AUC of 0.857 (95% CI, 0.622 0.973; p < 0.0009) with 78.57% sensitivity and 80% specificity (DCt cut-off value: ≤ 0.63348) (Fig 3A–3C). From the comparison of ERMs vs controls we obtained for miR-19b an AUC of 0.97 (95% CI, 0.755 1; p < 0.0001) with 90.91% sensitivity and 100% specificity (DCt cut-off value: >14.935); miR-24 had an AUC of 0.848 (95% CI, 0.595–0.973; p<0.0003) with 72.73% sensitivity and 83.33% specificity (DCt cut-off value: > 14.734); miR-142-3p showed an AUC of 0.933 (95% CI, 0.618 0.999; p< 0.0001) with 83.33% of sensitivity and 100% of specificity (DCt cut-off value: ≤ -1.535) (Fig 3D–3F). In the comparison of MHs vs controls, miR-19b showed an AUC of 1 (95% CI, 0.715 1; p< 0.0001) with 100% sensitivity and 100% specificity (DCt cut-off value: > 14.935); while, miR-24 had an AUC of 0.9 (95% CI, 0.576 0.997; p < 0.0001) with 80% sensitivity and 83.3% specificity (DCt cut-off value: > 14.734) (Fig 3G and 3H). We found no significant result for the comparison of ERMs vs MHs. These data suggested that the expression of vitreal miRNAs miR-19b, miR-24 and miR-142-3p was able to distinguish ERM and MH eyes from controls, but could not discriminate ERMs from MHs.

## Discussion

The results of this study show that in the VH of patients with MHs and ERM smicroRNAs have different levels of expression, and, in particular, miR-19b, miR-24 and miR-142-3p exhibit the most significant discriminative power compared to controls.

A decreased expression of miR-19b has been associated with the phenomena of fibrosis in liver and heart cells [34,35]. In addition, its decreased serum level has been reported in association with intestinal fibrosis [36].

Even the downregulation of miR-24 has been repeatedly associated with an increase of mechanisms of fibrosis in the heart [37,38].

Recent studies demonstrated that miRNAs derived from the miR-17-92 cluster (including miRNA-19b) directly modulate TGFβ signaling [39,40]. Also the miRNA-24 cluster has been reported to change TGFβ signaling through several pathways [41,42], suggesting a significant role of these miRNAs in TGFβ-mediated fibrogenesis.

Data in the literature show that overexpression of miRNA-19b and miRNA-24 may be a valuable therapeutic agent for TGFβ-mediated fibrosis [11].

Increased serum levels of miR-142-3p were associated with the presence and severity of scleroderma, an autoimmune disease that causes a progressive fibrotic tissue formation in the normal tissue architecture of various organs [36,43].

Furthermore, the increased levels of this microRNA in biopsies and lymphocytes have been associated with the presence of interstitial fibrosis as a result of kidney transplants [44].

It has also been shown that miR-142-3p modulates the production of cAMP and is involved in the regulation of macrophages and T cells [45]. Regulatory T cells lose their capacity to suppress immunological processes involving the kidney as suggested by the high levels of miR-142-3p in tissue samples of renal allografts [37]. Soltaninejad et al. found increased levels of miR-142-3p in allograft tissues of patients affected by interstitial fibrosis and tubular atrophy that is the major cause of renal transplant [37].

Downregulation of miR-19b and miR-24 and upregulation of miR-142-3p, already reported in the literature, are in agreement with the variations observed in our study in the VH of patients affected by MHs and ERMs and suggest that, as demonstrated in other pathologies, the different expression of these molecules is related to an increase of fibrosis, which is a characteristic feature of both MHs and ERMs [4–8].

Moreover, to understand the potential functional role of miRNAs differentially expressed in the VH, we performed a computational analysis on the network of the differentially expressed miRNA targets. Interestingly, among the functions significantly over-represented in both the vitreoretinal diseases, one of the most significant is related to TGF-β that has been linked to fibrogenesis [11].

To date, no approved treatments for fibrosis have been described. Several studies have described modifications in miRNA expression profiles during development of fibrosis that control wound-healing transcripts [46]. Wang et al. reported that *in vivo*, miR-24 could improve heart function and attenuate fibrosis in the infarct border zone of the heart two weeks after myocardial infarction through intramyocardial injection of Lentiviruses [34].

To the best of our knowledge, this is the first report describing a possible correlation between miRNAs and fibrotic phenomena that characterize patients affected by MHs and ERMs.

The main limitation of our study is the low number of patients and that the control group presented some vitreous abnormalities (symptomatic vitreous floaters). In particular, the low number of biologically independent replicates as well as the mixed presence of already-existing vitreous opacities in the control group might justify the wide dispersion highlighted in the expression of different miRNAs.

The source of miRNAs in the VH, as in other bodily fluids, could represent a critical point of debate. The most accepted hypothesis asserts that miRNAs are actively secreted in membrane-bounded-vesicles (i.e., exosomes), even if some studies suggest that most circulating miRNAs are in a non-membrane bound form, but rather assembled in ribonucleoprotein complexes (e.g., Ago2, or other RNA binding proteins) [47]. The hypothesis that circulating miRNAs are passively released into the extracellular environment as byproducts of dead cells has not been suitably untangled [24]. Moreover, miRNAs are rapidly degraded by RNases when secreted in blood without protection by vesicles or ribonucleoprotein complexes [19]. Accordingly, the real-time PCR dosage of circulating miRNAs resulting from physiologic and pathological flaking of the cells would be scarcely appreciable or extremely variable. Our data on miRNA disregulation in the VH exclude RNA contamination from the few cells floating in the vitreous (i.e., phagocytes, hyalocytes of Balazs) because VH samples were appropriately centrifuged to pellet and remove any circulating cells before RNA extraction (see Methods). Moreover, in our previous work we demonstrated that exosomes floating in the VH have miRNA expression profiles statistically related to those observed in total VH [26]. These data suggested that the concentration of circulating miRNAs in the VH could be mostly, but not exclusively, due to the molecular content of VH exosomes. For this reason, we believe that vitreal miRNAs, detected as being altered in MHs and ERMs, may be the result of a dysregulated signaling carried by exosomes secreted by the epithelial cells of the retina or from floating cells in the vitreous cavity. Disregulation of miR-19b, miR-24 and miR-142-3p, might be related to the pathological alterations that characterize patients affected by MHs and ERMs. The concentrations of these vitreal miRNAs also discriminated pathological eyes from controls, but they were not able to distinguish between MHs and ERMs. However, these results would suggest the possibility to exploit a possible ocular pharmaceutical RNA-based treatment against these differentially expressed miRNAs that might be administered to the patients affected by these slow-developing alterations, reducing invasive therapeutic approaches, such as vitrectomy.

## Supporting information

S1 File Ct raw data from Microrna expression profiling. Raw Ct data of TaqMan^®^ Array Microfluidic Cards A + B from vitreal samples of 4 macular holes (MHs), 4 epiretinal membranes (ERMs), 4 controls.
(XLS)
