# Correction: miRNAs in the vitreous humor of patients affected by idiopathic epiretinal membrane and macular hole

**DOI:** 10.1371/journal.pone.0176618

**Published:** 2017-04-20

**Authors:** Andrea Russo, Marco Ragusa, Cristina Barbagallo, Antonio Longo, Teresio Avitabile, Maurizio G. Uva, Vincenza Bonfiglio, Mario D. Toro, Rosario Caltabiano, Cesare Mariotti, Francesco Boscia, Mario R. Romano, Cinzia Di Pietro, Davide Barbagallo, Michele Purrello, Michele Reibaldi

The twelfth author's name is incorrect. The correct name is: Mario R. Romano.
